# The unmet needs in management, the treatment gap and the burden of migraine in Greece: a Delphi consensus and focus group study from patients’ perspective

**DOI:** 10.3389/fneur.2025.1558014

**Published:** 2025-02-24

**Authors:** Michail Vikelis, Georgia Kourlaba, Lia Barba, Konstantinos Bilias, Elena Barbalia, Argyro Solakidi, Dimitrios Trafalis, Katerina Lioliou, Sofia Zyga, Theodoros Karapanayiotides, Dimos-Dimitrios Mitsikostas

**Affiliations:** ^1^Headache Clinic, Mediterraneo Hospital, Glyfada, Greece; ^2^Department of Nursing, Faculty of Health Sciences, University of the Peloponnese, Tripoli, Greece; ^3^Greek Society of Migraine and Headache Patients, Athens, Greece; ^4^Hellenic Headache Alliance, Athens, Greece; ^5^Pfizer Hellas, Athens, Greece; ^6^Second Department of Neurology, Faculty of Health Sciences, School of Medicine, AHEPA University Hospital, Aristotle University of Thessaloniki, Thessaloniki, Greece; ^7^First Neurology Department, Eginition Hospital, Medical School, National and Kapodistrian University of Athens, Athens, Greece

**Keywords:** migraine, Delphi consensus, patients, burden, unmet needs, Greece

## Abstract

**Introduction:**

Migraine is a chronic, debilitating neurological disease affecting more than 1 billion patients, worldwide. Even though migraines are not life-threatening, they have profound effects on individuals, families, and society.

**Objective:**

The aim of this study was to describe patients’ perspectives on socioeconomic and humanistic burden of migraine, as well as the unmet medical needs in the clinical management of migraine, in Greece.

**Methods:**

A mixed study design of a Delphi panel and a focus group was conducted, in which 16 and 9 episodic migraine patients from the two Greek migraine patient associations participated, respectively. A structured questionnaire of 45 statements regarding burden of disease diagnosis, treatment and unmet needs was used to collect data at Delphi consensus panel. An open and balanced discussion with all participants took place, under the co-ordination of one moderator during the focus group. At each round of the Delphi panel, the percentage of participants who responded “Agree/Strongly Agree,” “Disagree/Strongly disagree” and “Neither Agree nor Disagree” was calculated. The consensus threshold was set at 70% of responses. A thematic analysis was performed for the focus group.

**Results:**

Consensus was achieved on 34 out of 45 statements. Thematic analysis revealed that patients face severe problems at work due to a lack of understanding and awareness of migraine burden from colleagues and employees, they are forced to modify their daily activities to avoid migraine attacks, and they experience prolonged diagnostic and treatment journey since they visit different medical specialties until they find the one who will be able to effectively help them manage their migraine. Additionally, they expressed concerns regarding the management of their disease due to patient-physician communication gap, dissatisfaction with traditional oral preventive therapies and lack of therapeutic options for older ages and in case of existence of specific comorbidities.

**Conclusion:**

Participants agreed that Migraine has a multifaceted impact on several aspects of patients’ lives. Patient-centered care, better training for healthcare providers, targeted therapies, and improved communication tools emerged as key factors in addressing the unmet medical needs of migraine sufferers.

## 1 Introduction

Migraine is a chronic, debilitating neurological disorder that affects approximately 1.1 billion patients worldwide (1), with an estimated global prevalence of 14–15% (2). According to recent data, national estimates show that 8% of Greek population suffer from migraine, whereas gender wise the prevalence follows global trends (3). Migraine prevalence is highest among individuals in their most productive years, with the number of cases increasing with age and peaking between the ages of 30 and 34 for both males and females, while the prevalence in women is estimated to be 2–3 times higher than men (1, 4). Recent data suggest that headache disorders, including migraine, are ranked 14th among global causes of Disability-adjusted life years (DALYs) for all ages in both genders (4).

Migraine may manifest itself by various and heterogenous symptoms, with headache being one of them (5). In general, it is characterized by recurrent episodes of moderate to severe headaches that can last up to 72 h, if left untreated. Migraine attacks may also be accompanied by nausea, vomiting, photophobia, phonophobia, and depressive symptoms (1). In some cases migraine may be accompanied by transient focal neurological symptoms that usually precede or sometimes accompany the headache phase and are the manifestations of the migrainous aura (6). In addition, migraine is divided, based on monthly frequency into chronic migraine (CM) and episodic migraine (EM) (7). The International Classification of Headache Disorders (ICHD-3) criteria define CM as headache on ≥15 days a month for more than 3 months, which, on at least 8 days/month, has migrainous features or respond to migraine-specific analgesics (6). On the other hand, EM is characterized by less than 15 headache days monthly, with 8–14 days being classified as high frequency, and less than 8 days as moderate/low frequency episodic migraine (7, 8). According to the most recent epidemiological study, the 1% of Greek adult population suffers from CM, and 7.1% from EM (3). Μigraine often coexists with other disorders, such as depression, anxiety, epilepsy, chronic pain and cardiovascular events (9). In 2019, the Hellenic Headache Society conducted a consensus on migraine diagnosis and treatment to raise awareness, support practitioners, and improve patient care in Greece (10). According to this consensus diagnosis is based on patient history and clinical examination (10). In addition, as per this consensus, mild migraines are treated with high-dose simple analgesics, while moderate to severe cases require triptans, non-steroidal anti-inflammatory (NSAIDs), or a tailored combination (10). Preventive treatments include beta-blockers, flunarizine, valproate, topiramate, and candesartan for episodic migraines, and botulinum toxin or monoclonal antibodies targeting the CGRP pathway (anti-CGRP) for chronic or high-frequency migraines after failed prior treatments (10). Migraine translates into an immense clinical and financial burden both for migraineurs and society. More specifically, several day-to-day activities of patients’ lives such as work, school, and social, as well as overall quality of life, are frequently substantially affected (7, 8, 11–15). From an economic point of view, headache disorders have been associated with staggering costs, with estimations reaching up to €173 billion in Europe, with approximately 90% being attributed to indirect costs (16).

Migraine may also have a profound impact on patients’ quality of life and the healthcare economy (7, 8, 11–17). Despite significant advances in the field of treatment, the disease is still associated with substantial unmet medical needs, mostly due to underdiagnosis and undertreatment (18). Over the past decade, several Greek studies have been conducted in order to assess the burden of the disease, the epidemiology, and patients’ preferences (3, 19–27). These studies had been conducted using structured questionnaires providing strong numerical data but not allowing the participants to share details of their testimonies, and generated data regarding the novel treatments that were introduced for the first time in Greece (19, 28).

However, given that the landscape in migraine treatment has been rapidly changing over the last few years, it is crucial to capture a current snapshot from the patients’ standpoint regarding the migraine-related issues that they face. In this context, a Delphi survey followed by a Patients’ focus group were conducted to depict patients’ perspectives on socioeconomic and humanistic burden of migraine, as well as the unmet medical needs in the clinical management of migraine, in Greece.

## 2 Methods

### 2.1 Study design and participants

To serve the objectives of the present study, a mixed design was selected. At first a modified Delphi consensus panel was conducted and then the findings of this panel were further elaborated through a patients’ focus group where they, through an open discussion, conveyed their personal experience of migraine. The Delphi method panel comprised three rounds over a period of 1 month (between March 20 and April 4, 2023). The patients’ focus group was held via videoconference lasting 3 h.

As for the Delphi panel, 20 members of the two active migraine associations in Greece were invited to participate. More specifically, the research team contacted the two Greek, migraine patient associations: the ‘Greek Society of Migraine and Headache Patients’ and the ‘Hellenic Headache Alliance.’ The Management Boards of these associations extended the invitation to 20 adult patients diagnosed with EM who were actively involved in their respective associations to voluntarily participate in the Delphi process. The selection of patients with EM in the study was based on the fact that it is the most common form of migraine according to epidemiological data, compared to CM (3). Additionally, although patients with EM may experience fewer than 15 migraine days per month, these episodes are significant enough to make them aware of the disease, thus making them more suitable for our study. Migraineurs who expressed interest in the invitation and provided their informed consent were recruited in the study, 13 members of ‘Greek Society of Migraine and Headache Patients’ and three members of the ‘Hellenic Headache Alliance’ (*n* = 16). Nine participants of the Delphi panel were randomly selected to participate in the focus group. All of them accepted the invitation and gave their consent to participation.

### 2.2 Data collection

As for the Delphi panel, a structured online questionnaire, developed in Qualtrics (Provo, USA) by a steering committee of three neurologists and an expert of research methodology, was used for data collection. The questionnaire, consisting of 45 statements, was developed based on data identified through extensive literature research in PubMed database and other scientific society websites. These statements were divided into five thematic sections: Burden of migraine (11 statements), Economic burden of migraine (5 Statements), Diagnosis (8 Statements), Treatment (13 Statements) and Unmet medical need (8 Statements). The questionnaire was sent using individualized, secure links delivered by e-mail, ensuring anonymity and encouraging honest responses. A 5-point Likert scale was used to measure participants’ level of agreement with each statement (“Strongly Agree,” “Agree,” “Neither Agree nor Disagree,” “Disagree,” and “Strongly Disagree”). Response rate was augmented by e-mail reminders. Participants’ email addresses were not recorded. To facilitate convergence, the voting process was conducted in multiple (three) rounds. Upon completion of the initial round, the data were processed, and participants were provided with the consolidated results. A second round of voting was conducted including the statements which did not achieve consensus in the previous round, taking into consideration the results of the first round. After reprocessing the data from the second, a third and final round was carried out.

As for the focus group, an open and balanced discussion by all participants took place, under the co-ordination of one person (moderator). During the focus group, the results of the Delphi panel were presented, and the participants were encouraged by the moderator to comment on these, especially for statements on which consensus had not been reached. More specifically, the moderator during the meeting used prompts or probes to engage participants in deeper discussion: (a) prompting patients to provide more detail (“I would like to turn on the microphones and discuss this finding,” “I would like to hear your point of view”), (b) encouraging the continuous dialog (“What do you think is the reason for lack of consensus on this particular statement?”), and (c) summarizing and paraphrasing participants’ narratives to avoid confusion. Through the focus group process, the research team attentively listened to notes of the key words and topics to ensure all the valuable insights were collected from the patients’ perspective. The discussion was recorded and then fully transcribed verbatim, with the consent of all participants. The full literal transcription of the meeting, and the field notes of the research team were all gathered to perform a qualitative analysis.

### 2.3 Data management and analysis

As for the Delphi panel, the percentage of participants who responded “Agree/Strongly Agree,” “Disagree/Strongly disagree” and “Neither Agree nor Disagree” was calculated at each round. The consensus threshold was based on the guidelines proposed by Hasson et al. for the Delphi technique, according to which, a consensus level of 51–80% is recommended depending on the research objective, available resources, and other relevant factors (29). Given that the primary objective of this study was to identify unmet medical needs in migraine management, rather than to provide guidelines, the consensus threshold was set at 70% of responses, focusing on borderline options (“Strongly Agree,” “Agree,” “Disagree,” “Strongly Disagree”) (29).

As for the focus group, a thematic analysis was conducted through the provided minutes, by grouping meaningful units that refer to similar issues or content until the main topics emerged. Two researchers independently examined the minutes, to identify the relevant content. As soon as each individual analysis was completed, meetings between the two researchers were held to compare the results obtained and then to combine them. In the case of differences in opinion, theme identification was performed based on consensus. Afterwards, the research team held joint meetings to show, combine and integrate the results of the analysis from each researcher. Final themes were identified through collective discussion among all research members. Qualitative software was not utilized for data analysis.

### 2.4 Ethics statement

Εthical approval of the study protocol was obtained from the Research Ethics Committee of the University of the Peloponnese. Prior their participation to the Delphi panel and focus group, all patients were required to provide written informed consent, after being thoroughly informed about the study’s purpose (29, 30). All the information obtained throughout the study cannot personally identify participants or their responses in this online survey (30). All research data was handled in accordance with the European and national regulations for the protection of personal data in scientific research.

## 3 Results

### 3.1 Delphi panel results

#### 3.1.1 Characteristics of Delphi panel participants

Table 1 describes the demographics of the Delphi panel participants (*n* = 16). All panelists were adults, with a mean age of 42.8 years old (SD 8.2). The majority of the participants were women (64.3%) and all participants were employed. In terms of educational background, over half of the participants (57.14%) had completed post-graduate/doctoral studies, while 21.43% had received higher education.

**Table 1:** Panelist demographics.

Parameter	Value (%)
Sex	
Female	64.3%
Male	35.7%
Age	
18–29	7%
30–40	21%
41–50	43%
51–60	29%
Highest educational background	
Elementary school	14.3%
Highschool- Professional school	7.14%
Higher educational institutions- Technical educational institutions	21.43%
Post-graduate, doctoral studies	57.14%
Professional background	
Self-employed	42.9%
Salaried employee	57.1%
Unemployed	0%
Student	0%
Retired	0%
Other	0%

#### 3.1.2 Humanistic and economic burden of migraine

Consensus was reached on twelve out of sixteen statements in this section, with five statements achieving 100% agreement in the first round (Table 2). No consensus was reached when patients were asked about experiencing a migraine-associated stigma and regarding health professionals’ incapability to fully understand the severity of the symptoms that people with migraine are experiencing. Moreover, consensus was not reached regarding the increased frequency of the visits to physicians and emergency hospital departments, and the frequent use of costly diagnostic tests.

**Table 2:** Results regarding Burden of disease (quality of life, social impacts, psychological impacts, work-related impacts).

Statements	Round	Level of consensus
Migraine significantly affects the quality of life of people suffering from the disease (31).	1st	100% Agreement
People suffering from migraine experience mood disorders, sleep disturbances, or anxiety in their life (49–51).	1st	100% Agreement
People suffering from migraine may be less productive at work (32).	1st	100% Agreement
In a migraine episode, people suffering from migraine may have needed to take sick leave from their job or be absent from professional obligations (50, 51).	1st	84.6% Agreement
People suffering from migraine may have experienced social stigma due to their disease (34, 35, 52, 53).	3rd	38.4% Agreement 38.5% Nor agree, nor disagree. 23.1% Disagreement
People suffering from migraine feel that others underestimate the severity of symptoms of their condition (34).	1st	77% Agreement
People with migraine feel that health professionals do not fully understand the severity of the symptoms they are experiencing.	3rd	61.5% Agreement 23.1% Nor agree, nor disagree 15.4% Disagreement
People with migraine experience significant problems in their social interactions during migraine attacks.	1st	100% Agreement
During severe migraine attacks, people suffering from migraine may be unable to perform daily activities and may be bedridden during a migraine episode (31).	1st	100% Agreement
People suffering from migraine may feel that burden their family and social environment.	1st	84.7% Agreement
People suffering from migraine often modify and adjust their life to avoid an impending migraine episode (54).	1st	92.3% Agreement
Migraine may delay the professional/economic progress of people suffering from migraine (55, 56).	3rd	84.6% Agreement
Part of expenses related to migraine treatment is due to buying multiple medications and dietary supplements (vitamins) that are not prescribed or covered for people suffering from migraine (55, 57).	1st	77% Agreement
Part of expenses related to migraine is spent on alternative methods to relieve symptoms, such as acupuncture, psychotherapy, homeopathy for people suffering from migraine (57).	1st	84.7% Agreement
People suffering with migraine frequently visit doctors of various specialties and hospital emergency departments due to their migraines (58).	3rd	61.5% Agreement 23.1% Nor agree, nor disagree 15.4% Disagreement
Because of migraines, people suffering from migraine have undergone numerous costly diagnostic tests.	3rd	30.7% Agreement 53.9% Nor agree, nor disagree 15.4% Disagreement

#### 3.1.3 Diagnosis

Regarding diagnosis, six out of eight statements garnered consensus among the panelists; two statements met with disagreement, while one achieved unanimous agreement (100%) (Table 3). No consensus was reached regarding the identification of the triggers that initiate a migraine episode and the association between the delay in diagnosis and the examination time spent by the physician.

**Table 3:** Results on diagnosis.

Statements	Round	Level of consensus
Most of the time, people suffering from migraine can identify what triggered a migraine episode.	3rd	53.8% Agreement 30.8% Nor agree, nor disagree 15.4% Disagreement
Most of the time, people suffering from migraine can usually recognize the symptoms of a migraine episode immediately.	1st	84.7% Agreement
It can take up too many years before people with migraine seek medical advice, as they have learned to live with their symptoms and manage them partially and ineffectively with simple analgesics (59).	1st	100% Agreement
The stigma and emotional burden experienced by people with migraine from the social environment reinforces their reluctance to seek treatment.	2nd	76.7% Disagreement
People with migraine are initially referred to other medical specialties before being referred to a specialist neurologist.	1st	84.6% Agreement
People suffering from migraine have difficulty fully describing their migraine symptoms to the treating doctor (60, 61).	3rd	76.9% Disagreement
The delayed diagnosis may be due to the limited time the treating doctor allocated during the visits of people suffering from migraine (18).	3rd	7.7% Agreement 61.5% Nor agree, nor disagree 30.8% Disagreement
The delayed diagnosis of migraine led to an increase of migraine episodes in people suffering from migraine (18).	2nd	92.3% Agreement

#### 3.1.4 Treatment

In this section, nine out of thirteen statements reached consensus in agreement, with two of them achieving 100% agreement (Table 4). Consensus was not reached regarding about the use of opioids for the management of a migraine attack, the delayed relief time of recommended medications during a migraine attack, patients’ compliance with their doctor’s instructions on pharmaceutical treatment, and patients’ decision to discontinue preventive medications upon improvement.

**Table 4:** Results regarding treatment of migraine.

Statements	Round	Level of consensus
For managing a migraine attack, people suffering from migraine usually use simple analgesics, or anti-inflammatory drugs, and then triptans (10).	1st	100% Agreement
People suffering from migraine during a migraine attack may use opioid analgesics, in order to manage it (62).	3rd	46.2% Agreement 46.2% Nor agree, nor disagree 7.7% Disagreement
People suffering from migraine use a lot of medications to manage the disease (63).	1st	100% Agreement
Therapeutic decisions made by the treating doctor should take into account patients’ preferences, lifestyle specifics (10, 64).	2nd	84.6% Agreement
Often, the drugs recommended for relieving a migraine episode take time to take effect.	3rd	61.5% Agreement 7.7% Nor agree, nor disagree 30.8% Disagreement
With timely administration of the medication, people with migraine find relief from migraine symptoms.	2nd	76.9% Agreement
People with migraines consider triptans as the most effective treatment for managing migraine attacks.	1st	77% Agreement
People with migraines prefer oral preventive treatments compared to injectable ones.	2nd	76.9% Agreement
People with migraines always follow their doctor’s instructions regarding the pharmaceutical treatment of migraines (e.g., dosage, timing of administration).	3rd	23.1% Agreement 53.9% Nor agree, nor disagree 23.1% Disagreement
People with migraines who are on preventive treatment may stop the therapy on their own when they see improvement.	3rd	38.5% Agreement 38.5% Nor agree, nor disagree 23% Disagreement
People with migraines, when they undergo preventive medical treatment, expect an immediate improvement of their symptoms.	1st	83.3% Agreement
Adherence to the treatment of people with migraines depends on how effective their communication with their doctor is.	1st	75% Agreement
People with migraines are experiencing high rates of dissatisfaction with oral preventive treatments.	3rd	84.6% Agreement

#### 3.1.5 Unmet medical need

A clear consensus was achieved regarding the existence of unmet medical needs, with seven out of eight statements aligning in agreement including two that reached unanimous agreement (100%) (Table 5). Consensus was not reached regarding whether a reduced number of medications would make adherence to migraine treatment easier.

**Table 5:** Results regarding unmet medical need of migraine.

Statements	Round	Level of consensus
Despite the wide availability of treatments, people with migraines are not very satisfied with their therapy (41, 42).	2nd	76.9% Agreement
Education of healthcare providers is required to achieve proper management of migraine (45).	1st	75% Agreement
There is a need for targeted therapies only for migraine.	1st	100% Agreement
There is a need for a treatment that could be used both as preventive therapy and for relief during a migraine attack.	1st	91.7% Agreement
There is a need for treatments whose therapeutic effect can cover the entire duration of a migraine attack.	1st	100% Agreement
There is a need for treatments that can be safely administered to patients with cardiovascular problems.	1st	91.7% Agreement
Adherence to the treatment of people with migraines would be easier if they had to take fewer medications.	3rd	38.5% Agreement 61.5% Nor agree, nor disagree
The use of aids (applications, patient decision aids, apps, e-diaries) by migraine patients, where they can easily record critical information about their migraine and share it with their clinical doctor during medical visits, would facilitate communication and optimize treatment outcomes (47).	1st	91.7% Agreement

### 3.2 Focus group results

The patients’ discussion during the focus group meeting was analyzed and three key themes emerged: (a) work and social life: lack of awareness and understanding; (b) limiting the impact of migraine on daily life; and (c) migraine management: late diagnosis, low treatment adherence and concerns about treatment. These three emerging themes were evident in the narratives retrieved from patients’ descriptions (Table 6).

**Table 6:** Testimonials from patients during focus group meeting.

Themes	Testimonials
Work and Social Life: Lack of awareness and understanding	**Lack of understanding**: *“It is very difficult for a migraine patient not only to ask for sick leave for their work but even to mention it as a problem.” (Patient 1, man); “It is true that migraineurs need to take sick leave, but they do not.” (Patient 1, man)*; “*It’s not within our family (understood) let us not have illusions. How would it be understood by the other people?” (Patient 3, woman); “We do everything in our power so that they do not even notice it within our family, even my own husband, I even hide it from my own children because I do not want to keep saying I have a headache today, leave me alone, I’m not well*” *(Patient 2, woman).* **Lack of awareness**: “*You cannot say 20 times a month, ‘I cannot come to work because I have a migraine,’ or ‘I want to take leave because I have a migraine,’ because for people who do not experience it, migraine is ‘how can you be like this for a headache? Take a painkiller’.” (Patient 2, woman); “I believe there is not proper communication among people regarding what really happens with migraine sufferers” (Patient 2, woman)*. **Stigma and internalized stigma**: *“The stigma is a very intense term.” (Patient 5, woman); “We may understand the word ‘stigma’ differently. The word ‘stigma’ is a heavy word. It’s a conversation that I would say carries a dose of denial within it.” (Patient 1, man); “Stigma is a heavy expression, but to understand and experience stigma in my workplace means that I create a problem in my work to receive the stigma. Typically, as migraine sufferers, as I mentioned in the previous question, we do not allow or communicate this thing. That is, even though we might wake up in the morning with a very intense migraine, we’ll delay going to work until the painkiller kicks in, but we’ll still go and work normally, and we will not communicate it so as not to cause a problem at work. Consequently, our colleagues might marginalize us, label us, and we would experience whatever stigma there may be.” (Patient 2, woman); “We become tiresome to our environment if day after day we say I have a headache” (Patient 2, woman).* **Embarrassment/Ashamed**: *“Ι think a large percentage of migraine patients do not talk about it. […] they do not say, ‘We’re migraineurs.’ I do not know why; are they afraid? Do they consider it shameful? Do they view it as such a big problem? I do not know the reason. The result, of course, is that if you do not say it, others do not know, so you are not stigmatized for being a migraine sufferer because they do not know.” (Patient 3, woman); “They* (*Migraine sufferers*) *do not want to communicate it because they perceive it will become a burden, they’ll become bothersome, they will not be believed. Many times, they feel guilty, thinking, ‘Again, I’ll say I have a headache, again I’ll say I cannot work.’ So, they hide it” (Patient 2, woman).*
Limiting the impact of migraine in daily life	**Daily live activities**: *“Our lives are full of don’ts and should not. We automatically forbid ourselves anything that might cause an attack” (Patient 2, woman)*. **Triggering mechanisms**: *“The triggering mechanisms are different for each individual.” (Patient 1, man); “When each of us knows more or less certain things that they have seen to be burdensome for themselves, they are forced not to completely avoid them. Okay, then we go to the other extreme of having to lose many pieces of our social life, which is not feasible and should not even happen.” (Patient 4, woman); “[…] there is not always a triggering mechanism.” (Patient 1, man); “[…]we are just not all sure if we have finally figured out what caused our migraine. Because sometimes we take it for granted that it was a late night’s sleep that triggered our migraine and other times we have stayed up all night without having a migraine or we have a migraine when we have had a good night’s sleep.” (Patient 1, man) “Yes first of all I think that to generally identify the triggering factors takes a long time at least in most patients, these factors change over decades and at certain stages of life” (Patient 6, woman).*
Migraine management: Late diagnosis, low treatment adherence and concerns about treatment	**Late diagnosis – Self-medication**: *“Each patient has their own judgment of what constitutes a rapid crisis treatment.” (Patient 1, man); “I because I am diagnosed with migraine I know the simple analgesics some of which I used from a young age until I suddenly learned from a pharmacist about the triptans and I started using the triptans with the encouragement of the pharmacist before I went to the neurologist where he then gave me the okay to use the triptans*” *(Patient 2, woman).* **Late diagnosis – Seeking the appropriate medical expert**: *“Regarding frequent visits to various doctors, yes, I believe all migraine sufferers start with a series of consultations with different specialties until they find the right doctor, if they are lucky.” (Patient 2, woman); “Someone may even go to an ophthalmologist, but the neurologist is usually the last in line they will visit. However, this is changing now.” (Patient 7, woman); “Before I ended up seeing specialized neurologists, I had probably been through all the medical specialties that exist. No one told me, ‘You know, maybe you should see a neurologist. I’m a pathologist, and you need to see a neurologist.’” (Patient 1, man); “As the severity and frequency of migraines increase, individuals often begin seeking solutions from doctors they have regular communication with, such as their gynecologist or primary care physician. As the condition worsens, they may intensify their search for solutions.” (Patient 7, woman); “I had to go to several neurologists to convince someone that I needed prophylactic treatment” (Patient 6, woman).* **Late diagnosis – Necessity of effective migraine management**: *“The journey and search for effective treatment peaks at ages 35 to 45 and 50. […] things become much more challenging for people in those ages, resulting in migraine becoming a serious issue that causes many problems” (Patient 1, man)*. **Late diagnosis – Physician/ medical expert understanding**: *“The important thing is that migraine patients are often psychologically vulnerable. Therefore, I believe that the character of the migraine sufferer plays a significant role in medical treatment.” (Patient 3, woman); “The years of suffering, the frequency, and overall experience one has are the most important aspects in terms of understanding from the professionals’ side. For some of us whose journey with migraines began decades ago, I simply want to say that understanding seems to be something that has improved over the last decade, I would say, compared to 20 years ago” (Patient 6, woman)*. **Late diagnosis – Unclear symptom comprehension**: *“[…]the level of understanding depends on the doctor’s specialty. Not all migraine patients go to specialist neurologists who know exactly what the problem is and know the extent of the symptoms and dysfunction caused by the migraine.” (Patient 1, man); “The difficulty is when it’s not a specialist doctor,* i.e.*, if you have to go to a general practitioner, it’s difficult for him to understand what the aura you are feeling is” (Patient 3, woman)*. **Late diagnosis – Diagnostic tests**: *“So, we are always talking about the specialized doctor who knows about this. They will not ask you to get a brain MRI because a brain MRI does not show anything. So, it’s pointless.” (Patient 1, man); “Migraine cannot be certified through MRI scans. Therefore, we cannot understand why the National Organization for Healthcare Services Provision requests MRI scans and rejects requests; another reason could be found.” (Patient 1, man); “Cases from the past involved older practices where older doctors might have requested MRIs, angiographies, or even repeated MRIs every five years” (Patient 6, woman)*. **Low treatment adherence – Treatment dissatisfaction**: *“One reason we see this percentage here is high because all these treatments, which are oral, were not exclusively designed for migraines” (Patient 1, man)*. **Low treatment adherence – Preventive treatment**: *“People prefer to take something once a month and not worry about it, which is why monoclonal antibodies, among other treatments, have been highly successful in this regard. They make medication adherence more consistent.” (Patient 1, man); “They consider that since they already feel better, they can stop it. So, they stop taking the medication.” (Patient 3, woman); “What improvement means for each person is relative and not easy to answer.” (Patient 1, man);“What the patient expects is for the results to be immediate.” (Patient 2, woman); “I think the medicine and what advice the doctor will give plays a role here. That is, the doctor knows when you will see results from a prophylactic medication” (Patient 2, woman)*. **Low treatment adherence – Follow up visits**: “*So, I think that on average, a visit to the neurologist every three months is required to evaluate the situation and adjust medication accordingly.[…] Even if someone goes to the neurologist every three months, I believe that once someone decides to start preventive therapy, it is essential to monitor the course of that specific treatment properly” (Patient 1, man)*. **Low treatment adherence – Aids for effective monitoring**: “*The issue with these applications, and unfortunately many times the developers who create them struggle to understand, is that they should be as simple and user-friendly as possible.” (Patient 1, man);“[…] if someone has the willingness and time to consistently fill them out, it can truly pay off in the long run. It becomes a valuable database on their phone that they can share with their doctor, yielding useful insights” (Patient 1, man)*. **Agony for new therapies**: *“So, we have a significant concern among members of the association who send us many messages. They are mature individuals, aged 62 or 63, wondering what will happen when they turn 65. They worry about whether their treatment will be discontinued and what they will do or take in such a scenario” (Patient 1, man);* “*I share the same anxiety because I am soon reaching the age of 65, and I experience over 20 crises per month” (Patient 3, woman)*.

#### 3.2.1 Work and social life: lack of awareness and understanding

Patients emphasized the need for sick leave during migraine attacks due to inability to fulfill professional obligations. However, lack of understanding from their colleagues and managers/employers discourages their absences from work. They emphasized that this lack of understanding from their workplace might stem from lack of awareness about migraine. In addition, patients noted that they avoid communicating their disease and self-isolate in order not to be a burden for their friends and family. On that basis, migraine patients do not communicate their condition, resulting in their self-isolation and self-stigmatization. Additionally, they commented that, as a term, the word “stigma” is perceived with a strong negative connotation. Some patients admitted feeling ashamed about their disease.

#### 3.2.2 Limiting the impact of migraine in daily life

Migraineurs live in fear of another migraine episode. As such, they modify their daily activities to avoid even a migraine attack per month, as even this will be a benefit to them. Patients mainly discussed the triggering mechanisms and how they widely vary among individuals. Some patients, despite acknowledging the existence of triggering mechanisms in migraine, noted that these mechanisms are not a sufficient and necessary condition for the existence or avoidance of a migraine attack. However, patients seem to believe that with appropriate treatment, they can stop modifying their lives to such a large extent and live more freely.

#### 3.2.3 Migraine management: late diagnosis, low treatment adherence and concerns about treatment

During the focus group, the patients reported various factors leading to the delayed diagnosis of migraine and consequently to its appropriate treatment. Many patients admitted that they have been self-medicating with over-the-counter drugs and pain relievers for a long period to control their disease, before seeking professional medical advice. Moreover, they mentioned that it takes them a long time to find the appropriate medical care, since they visit different medical specialties until they find the one who will be able to effectively help them manage their migraine. The need for effective migraine management peaks during middle age, resulting in the most persistent seeking of medical care. Physicians who are not specialized in Migraine/Headache disorders, seem to have inability to fully comprehend migraine patients and the severity of their symptoms seems to attribute to delay diagnosis, as well. Participants highlighted the need for further training of Physicians/Neurologists on headache disorders to increase their awareness. However, a noticeable improvement in physicians’ understanding of migraine during the last years was pointed out by patients.

Moreover, participants mentioned that they are subjected to various diagnostic tests when they wander between different medical specialties looking for their diagnosis, while a neurologist/migraine specialist can make a diagnosis based only on the patient’s detailed medical history. Many patients mentioned that clinical practice that involves costly diagnostic tests is considered outdated. However, they noted an interesting paradox in the Greek healthcare system; the fact that certain high-cost diagnostic tests (e.g., magnetic resonance imaging-MRI) have occasionally been requested for prescription and reimbursement of newer, more targeted to migraine treatments. Moreover, patients have actively reached out to the National Health Service Provider Agency (E.O.P.Y.Y.) to address and eliminate this practice.

During the discussion patients stated that there were high levels of dissatisfaction with the traditional oral preventive therapies because some of them were prescribed as off-label treatments. The patients’ expectation of immediate reduction of migraine attacks leads to disappointment that the desired outcome is not achieved and then to lower adherence to preventive migraine treatments. Moreover, patients’ non-adherence to preventive treatment of migraine and its early discontinuation may also be driven by the relief of their symptoms. In this context, patients emphasized the value of effective patient-physician communication in preventive treatment adherence. Moreover, patients noted that regular monitoring with neurologist visits every 3 months and adjustment of medication are crucial aspects of migraine management that might result in increased treatment adherence, particularly for those receiving preventive therapy. In addition, they highlighted the need for physicians to communicate treatment-related information clearly and comprehensibly. Furthermore, they highlighted the importance of easy-to-use aids, − such as applications, patient decision aids, apps, e-diaries, − in creating a patient-specific database from which both the physicians and patients can draw valuable conclusions. Finally, patients expressed their concern/agony about their therapeutic alternatives at older age and in case they suffer from specific comorbidities.

## 4 Discussion

This study aimed to capture patients’ views and experiences to highlight the socioeconomic burden and the unmet medical needs regarding the clinical diagnosis and management of migraine, in a Greek, real-world setting. To the best of our knowledge, although, there have been Greek studies in the past which analyzed quantitatively patients’ preferences and satisfaction with treatment as well as the burden of disease, this is the first one to describe the experiences of migraineurs through a qualitative analysis (3, 19–21, 28).

Our results regarding patients’ day-to-day activities are in line with those of a previous Greek study, highlighting the need for patients to modify their lives during a migraine crisis or in order to avoid it (3). A plethora of studies have shown that migraineurs often find it difficult to carry out daily tasks, including substantial disruptions in their work efficiency, due to migraine episodes (31–33). In our findings, it was clear that despite the need for sick leave during migraine attacks, migraneurs rarely ask for sick leave, possibly due to social stigma from their colleagues and employers (3, 34, 35). There is evidence supporting that migraine patients may suffer from internalized stigma, a psychological and cognitive effect resulting from society’s negative perception of the disease (34). In our study, while patients emphasized that they felt underestimated by their social and family environment and struggled to communicate their disease, the social stigma associated with migraine was not fully expressed by them. This discrepancy, between our study and other published results could be attributed to the existence of negative connotations associated with the Greek term for “stigma.” Moreover, another possible explanation may be associated with the fact that patients tend to avoid stigmatization by bypassing any health-related discussion to avoid negative perceptions, but they are self-stigmatized. An Italian Delphi study focused on women with migraine, including patients in its panel, revealed that they also experience a high burden and are significantly impacted by stigma, which affects their well-being (36).

Our research revealed that Greek patients are self-medicated by receiving analgesics for years before seeking professional medical advice. This finding coincides with the observations published by Davies et al., according to which patients suffer between 6 and 10 years prior to seeking medical advice, resulting in delays receiving a formal migraine diagnosis (37). Late diagnosis, either due to patients’ negligence or due to the involvement of multiple physicians before referring to a headache specialist, remains a major challenge in migraine management. In the Italian Delphi study, highlighted that women with migraine often misinterpret their symptoms as unrelated to a disease, causing delays in seeking medical support (36). Thus, it is crucial to streamline the diagnostic process and raise awareness both among patients and healthcare providers.

Another topic of great importance in the treatment of migraine is patient’s adherence. Patients’ adherence is a multidimensional phenomenon (32). In line with previous studies, our study also stressed the importance of shared patient-physician decision-making in the course of their treatment (38, 39). In addition, similar to other published data (40), our study revealed that effective patient-physician communication has a significant impact on the patient’s adherence to the prescribed treatment regimens. These results illustrate the need to develop a patient-centered approach to establish relations of mutual trust between the patients and the healthcare systems and thus promote clinical decisions that will enhance treatment optimization.

Finally, from a treatment standpoint, our findings mirror the diverse and nuanced nature of the current therapeutic armamentarium for migraine. Overall, in accordance with existing evidence (41, 42), a high degree of dissatisfaction with traditional treatment options, was highlighted despite the plethora of available choices. Indeed, several migraine medications have safety profiles that are not always favorable, multiple interactions, several contraindications and age restrictions (43, 44). Furthermore, the need for specialized education by health care providers that has been previously reported, was also illustrated in our study (45). Undeniably, access to novel targeted therapies has revolutionized the landscape of migraine treatment, due to their efficacy and favorable safety profiles (3). The latter was also portrayed in our results. However, the need for developing safe and concurrently effective agents, especially for patients suffering from comorbidities such as cardiovascular diseases and people older than 65 years old, is still pending (32). According to a Dutch Delphi study with open-ended questions conducted among migraine patients, the primary concerns expressed by patients were the need for medications to act faster, relieve pain at an earlier stage, restore functionality quickly, and prevent recurrence (46).

In terms of improving treatment outcomes, our study showed that the incorporation of technology could facilitate seamless communication between patients and their clinicians. Existing evidence shows that the use of digital tools enables patients to easily record and share migraine-related information during medical visits, thus improving the quality of care and promoting better outcomes (47). However, while the available tools are promising for diary keeping, their daily use can be burdensome, often leading to reduced adherence or abandonment.

This study highlights several unmet needs that should be emphasized during clinical visits. Effective communication between patients and physicians is crucial for improving treatment adherence and patients’ satisfaction. Additionally, better education of healthcare providers on migraine management could reduce diagnostic delays and improve care. Finally, addressing patient dissatisfaction with traditional treatments and discussing newer, targeted therapies should be a priority in clinical practice to optimize health outcomes. Our study limitations are directly associated with the nature of the Delphi panel and focus group methodology. Specifically, limitations include the difficulty of generalizing the results to a wider population, due to small sample size and selection criteria of panelists (members of patients’ associations with EM), which is characteristic of the Delphi technique (48). The selection of patients with EM for the study was based on epidemiological data showing that it is the most common form of migraine compared to CM, and despite experiencing fewer than 15 migraine days per month, their episodes are significant enough to raise awareness of the disease, making them suitable for our study; however, this may limit the extrapolation of the results to the broader migraine population. However, these results offer important preliminary insights into the unmet needs and challenges faced by migraine patients, which may guide further research and clinical practice. Future studies should aim to recruit larger, more diverse, and representative patient populations to validate these findings. While in the case of Delphi panel entering data into an online platform is considered an advantage, it might also cause inconvenience for some panelists (48). Moreover, during the Delphi rounds, participants were unable to rephrase the statements, make comments, or request clarifications. This restriction may have led to misunderstandings or misinterpretations of specific statements, which in turn contributed to failure to reach consensus. In addition, despite the consent of participants to take part in the open discussion during the focus group, there was concern that they did not feel comfortable fully expressing themselves in front of an audience. However, this study offers an initial reference point to further design and conduct more focused and populated consensus studies in our country, as well as contributing to a deeper understanding of the perspectives of patients.

## 5 Conclusion

This study highlights migraine’s multifaceted impact on patients’ daily activities, productivity and overall quality of life. Moreover, our results underline the challenges associated with timely diagnoses and effective treatment. Patient-centered care, better education for healthcare providers, targeted therapies and improved communication tools emerged as key factors in addressing unmet needs of migraine patients. Continuous recording of such information so that it is taken into consideration both by physicians and health policy decision makers is essential. It is important to promote patients’ involvement in studies that incorporate their ideas or address their concerns, as well as improved implementation of research findings because inclusive approach ultimately leads to improved quality of care.

## Data Availability

The raw data supporting the conclusions of this article will be made available by the authors, without undue reservation.
